# Annotations

**Published:** 1906-05-26

**Authors:** 


### ANNOTATIONS.

### An Exhibition of Sweated Industries.

As an example of instructive realism few experiences can compare with the Exhibition of Sweated Industries which was opened by Princess Henry of Battenberg on May 10. Here in all their horrid nakedness are to be seen some of the methods by which unorganised labour, ground down by unrestricted competition, struggles to secure a livelihood in a community professedly civilised and religious. The factory worker, partly as a result of combination and partly under the influence of legislation, enjoys at least many of the conditions necessary for physical and moral wellbeing; but the women who work at home are in many industries the victims of ruthless sweating. According to Professor George Adam Smith, who presided at the opening of the Exhibition, very few home workers can make twopence per hour; the average rate is indeed about one penny, and practically all of them work under the constant fear of a reduction of wages or a loss of employment. Further, it would seem that in some of these occupations—in shirt-making, for example—the conditions, so far from improving, are moving in the opposite direction, and the rate of pay has in recent years distinctly declined. The lines made immortal by the genius of Hood would appear to be as true to-day as when they were originally written, for at the existing rate of pay it is possible that by working at shirt-making for 17 hours a day a woman may realise the magnificent weekly wage of twelve to fourteen shillings. All this bears obviously on questions of individual health and upon sanitary conditions and regulations. But what is desired particularly by the organisers of the Exhibition is the education of public opinion and the quickening of the public conscience. That there must be something wrong in a social organisation which permits so revolting a state of matters to continue all will admit. The hope is that the latest method of pressing the wrong on public attention will succeed in suggesting a remedy.

### Education by Permeation.

In a paper under this title, Canon Barnett emphasises the fact that we are as largely educated unconsciously as we are consciously, "careless acts, manners, the pictures in a room, story-books, a chance paragraph in a newspaper," all may have lasting effects upon the bent of our minds and of our character. For the same reason a teacher educates as much by his manners as by his facts. Canon Barnett also lays stress upon the unconscious influence of university settlements. The subject is an interesting one. How much, it may be asked, is "education by permeation" due to faculties of intelligent observation and how much to sensitiveness which unconsciously feels rather than observes? No doubt in most people the two are combined, but in one person intelligent appreciation takes a higher place than feeling, while in another the reverse is the case. In this connection it may be asked what is the faculty we sometimes hear of as the "instinct" of a physician? Is it not that he has almost unconsciously absorbed indications of disease or phases of disease and can tell the inquirer, to use a common expression, what he feels to be the condition, yet may not be able to give clear reasons for his opinion. Such an explanation of so-called instinct does not, however, imply that this attitude of mind or feeling is by itself a satisfactory quality. Impressions should, if possible, be always analysed and reasons discovered for the conclusion formed in the mind. From another aspect, to which Canon Barnett refers, the social aspect, those accustomed to deal with the sick should acquire knowledge by permeation more, perhaps, than any other section or class of the community. They may learn much of disease, but they should learn still more of human nature.

### Ringworm in Schools.

Some of the petty annoyances of life may occasion more trouble than serious ills. Ringworm, by reason of its infectious character and the resistance it often presents to treatment, may transform children for the time being into nuisances. The teachers cannot have them at school and the parents do not want them at home, so they are sent out to pick up what education they can in the streets. According to Sam Weller's father this is the very best of all forms of education, but even Sam Weller when he decided to write a love-letter found that the streets did not provide all that was necessary. That children should be prevented from attending school, in these days, at least, cannot be considered desirable. The number of children absent from school on account of ringworm is very considerable. It is estimated that one-ninth of the absence caused by infectious illness is due to this disease. This large percentage is due mainly, not to the number of cases of ringworm, but to the length of time many of the cases last. Not infrequently ringworm may resist all ordinary methods of cure for two years or even longer. The only treatment that will with certainty cure the disease quickly is the $x$-ray treatment. By this method, in the majority of cases, the area affected rapidly loses its power to spread locally or to infect others, since the diseased hairs are destroyed and fall out, and the child is usually enabled to return to school in less than three months from the time of onset of the treatment. In order to combat the trouble occasioned by ringworm in the schools under its control, the Croydon Borough Council has, we understand, decided to provide means for employing the light apparatus. The treatment will no doubt include skilled medical supervision. Although the $x$-ray treatment is undoubtedly efficient, employed without due care, not only the diseased hairs are removed but the healthy may be killed over the area exposed to the rays, and permanent baldness be the result. Want of knowledge is serious, but there may be occasions when want of hair may be considered still more serious. However, such an unfortunate sequel may be said virtually never to occur where there has been careful attention to necessary detail, and the Croydon Borough Council has probably set an example which will be followed in other parts of the United Kingdom.

